# Molecular Characterization of Antigen-Peptide Pulsed Dendritic Cells: Immature Dendritic Cells Develop a Distinct Molecular Profile when Pulsed with Antigen Peptide

**DOI:** 10.1371/journal.pone.0086306

**Published:** 2014-01-27

**Authors:** Amy X. Yang, Numju Chong, Yufei Jiang, Jennifer Catalano, Raj K. Puri, Samir N. Khleif

**Affiliations:** 1 Tumor Vaccines and Biotechnology Branch, Division of Cellular and Gene Therapies, Center for Biologics Evaluation and Research, Food and Drug Administration, Bethesda, Maryland, United States of America; 2 Vaccine Branch, National Cancer Institute, Bethesda, Maryland, United States of America; 3 Cancer Center, Georgia Regent University, Augusta, Georgia, United States of America; Tulane University, United States of America

## Abstract

As dendritic cells (DCs) are the most potent professional antigen-presenting cells, they are being tested as cancer vaccines for immunotherapy of established cancers. Although numerous studies have characterized DCs by their phenotype and function, few have identified potential molecular markers of antigen presentation prior to vaccination of host. In this study we generated pre-immature DC (piDC), immature DC (iDC), and mature DC (mDC) from human peripheral blood monocytes (PBMC) obtained from HLA-A2 healthy donors, and pulsed them with human papillomavirus E7 peptide (p11-20), a class I HLA-A2 binding antigen. We then characterized DCs for cell surface phenotype and gene expression profile by microarray technology. We identified a set of 59 genes that distinguished three differentiation stages of DCs (piDC, iDC and mDC). When piDC, iDC and mDC were pulsed with E7 peptide for 2 hrs, the surface phenotype did not change, however, iDCs rather than mDCs showed transcriptional response by up-regulation of a set of genes. A total of 52 genes were modulated in iDC upon antigen pulsing. Elongation of pulse time for iDCs to 10 and 24 hrs did not significantly bring further changes in gene expression. The E7 peptide up-modulated immune response (KPNA7, IGSF6, NCR3, TREM2, TUBAL3, IL8, NFKBIA), pro-apoptosis (BTG1, SEMA6A, IGFBP3 and SRGN), anti-apoptosis (NFKBIA), DNA repair (MRPS11, RAD21, TXNRD1), and cell adhesion and cell migration genes (EPHA1, PGF, IL8 and CYR61) in iDCs. We confirmed our results by Q-PCR analysis. The E7 peptide but not control peptide (PADRE) induced up-regulation of NFKB1A gene only in HLA-A2 positive iDCs and not in HLA-A2 negative iDCs. These results suggest that E7 up-regulation of genes is specific and HLA restricted and that these genes may represent markers of antigen presentation and help rapidly assess the quality of dendritic cells prior to administration to the host.

## Introduction

Human dendritic cells (DCs) are the most potent antigen presenting cells. A relatively small number of DCs have a capacity to render a potent immunologic response against pathogens or other antigens, including tumors [1]. The most common classes of DCs are myeloid and plasmacytoid (or lymphoid). Myeloid DCs can be divided into at least two subsets: myeloid DC-1 which is a major stimulator of T-cells; and myeloid DC-2 which is a rare type and functions in fighting wound infection. Plasmacytoid DCs, having a plasma cell-like morphology, could play a major role in adaptive immunity as well as innate immunity [2]. A number of articles have been published on the characterization and use of DCs, peptide- or protein-pulsed DCs to treat patients with different types of cancers [3], [4].

DCs can be generated by culture of peripheral blood monocytes (PBMC) or bone-marrow derived mononuclear cells in the presence of a variety of growth factors. Granulocyte-macrophage colony-stimulating factor (GM-CSF) and interleukin 4 (IL-4) have been most widely used to generate immature DCs (iDC) [5]. Mature DCs (mDC) are generated with different maturation cytokines, e.g., tumor necrosis factor alpha (TNFα), CD40 ligand (CD40L), lipopolysaccharide (LPS), interferon-γ (INFγ), and cytokine cocktail [6], [7], [8]. In addition, some clinical trials have also used antigen-pulsed PBMC that are short-term cultured only with GM-CSF gaining some phenotype of DCs [9]. These cells are termed pre-immature DCs (piDC). piDC, iDC and mDC are predominantly identified by phenotypic expression of CD11c, CD80, CD83, CD86 and HLA-DR and down modulation of CD14 expression. A large number of studies have reported on the gene expression changes during differentiation from monocytes to iDCs and mDCs using different stimulation and different microarray platforms [10], [11], [12], [13], [14], [15], [16]. However, few studies have examined the role of antigen pulse on gene expression changes in different activation states of DCs, and identified biomarkers of activation that can be rapidly assessed for quality testing of antigen pulsed dendritic cells.

In this study, we have investigated transcriptional biomarkers of differentiated DCs and gene expression changes in piDC, iDC and mDC after pulsing with a Major Histocompatibility Complex (MHC) class 1 antigen peptide. We used a 10-mer peptide (human papillomavirus16 E7p11-20), which is frequently used in clinical trials [17]. We discovered that when different types of DCs were pulsed with the antigen-peptide, iDCs showed molecular changes but not mDCs. These results suggest that iDCs may efficiently uptake and process peptide antigen when pulsed *in vitro*, and that the identified genes could be used for assessment the quality of antigen pulsed DCs. Furthermore, the information obtained from such studies may provide insights in the understanding of the process of peptide-antigen uptake and presentation by dendritic cells.

## Materials and Methods

### Isolation of monocytes and generation of dendritic cells in vitro

Human monocytes from nine human leukocyte antigen (HLA) -A2, and two non-HLA-A2 healthy donors were obtained from the NIH Blood Bank. The monocytes were cryopreserved in liquid N2 until use. To generate dendritic cells, the cryopreserved monocytes were thawed, put in tissue culture plate for 4 hrs at 37°C. The non-adherent cells were removed by washing with phosphate buffered saline (PBS). The adherent cells were cultured in complete RPMI 1640 medium (Mediatech, Inc. Manassas, VA) supplemented with human GM-CSF (1000 U/ml, BD Biosciences, San Jose, CA) for two days to generate pre-immature dendritic cells (piDC) or with human GM-CSF (1000 U/ml) and IL-4 (50 ng/ml, BD Biosciences) for 4 days to generate immature dendritic cells (iDC), or with human GM-CSF (1000 U/ml) and IL-4 (50 ng/ml) for 4 days and additional 2 days with TNFα (20 ng/ml, BD Biosciences) and CD40 ligand (200 ng/ml, InvivoGen, San Diego, CA) to generate mature dendritic cells (mDC) (**Table 1**). Two donors (D1 and D2) were also matured by culturing 2 days with a cytokine cocktail, which contained IL-1β (10 ng/ml, BD Biosciences), IL6 (10 ng/ml, InvivoGen), TNFα (10 ng/ml) and PGE_2_ (10^−7^ M, Sigma-Aldrich, St. Louis, MO).

**Table 1:** RNA sample source and differentiation conditions.

Sample Name	Description	Culture Conditions	Donor
G1/piDC	pre-immature DC	GM-CSF 2 d	4
G2/piDC-E7	pre-immature DC	GM-CSF 2 d, E7^a^ 2 h	4
G3/iDC	immature DC	GM-CSF+IL-4 4 d	7
G4/iDC-E7	immature DC	GM-CSF+IL-4 4 d, E7 2 h	7
G5/mDC	mature DC	GM-CSF+IL-4 4 d, TNFα+CD40L 2 d	4
G6/mDC-E7	mature DC	GM-CSF+IL-4 4 d, TNFα+CD40L 2 d, E7 2 h	4
G7/mDC	mature DC	GM-CSF+IL-4 4 d, Cocktail^b^ 2 d	2
G8/mDC-E7	mature DC	GM-CSF+IL-4 4 d, Cocktail^b^ 2 d, E7 2 h	2

a E7 antigen peptide_11–20_, encoded by oncogene E7 of human papillomavirus (HPV) was used at a concentration of 5 ug/ml;

b Cocktail contains TNFα, IL-1β, IL6, and PGE_2_.

### Human papillomavirus (HPV) E7 peptide (p11-20) pulsing

A ten amino acid (p11-p20) HPV oncogene E7 peptide (YMLDLQPETT), and a 13 aa synthetic, non-natural pan HLA-DR-binding Epitope (PADRE) [(D)alanine -K- (L)cyclohexylalanine -VAAWTLKAA- (D)alanine], were obtained from NIH Pharmacy Department. Endotoxin assay was done using Pierce LAL Chromogenic Endotoxin Quantitation Kit (ThermoScientific Cat# 88282). There were no detectable endotoxins in the peptide solution (<0.1 EU/ml as the limit of detection of the kit is 0.1 EU/ml). To investigate the effect of peptide pulsing on gene expression of human dendritic cells, piDC, iDC and mDC generated from monocytes of four HLA-A2 donors (D1–D4) were incubated with 5 µg/ml peptide at 37°C for 2 hrs. For the time course experiment, iDCs generated from monocytes of additional 3 HLA-A2 donors (D5–D7) were incubated with the same E7 peptide for 2 hrs, 10 hrs and 24 hrs. To investigate whether the E7p11-20 stimulation is HLA-A2 specific, iDCs derived from two HLA-A2 donors and two non-HLA-A2 donors (D8–11) were pulsed with E7 peptide and PADRE peptide.

### Flow cytometry analysis of DC

To evaluate the phenotype of three stages of dendritic cells, fluorescein isothiocyanate (FITC) labeled antibodies specific for CD11c (eBioscience, San Diego, CA), CD14 (BD Biosciences, San Jose, CA), CD80 (BD), CD83 (BD), CD86 (BD) and HLA-DR (BD), as well as isotype control mouse IgG1 (eBioscience) and IgG2a (eBioscience) were used to measure the surface markers as described by the Manufacturer (BD). In brief, the cells in flow cytometry staining buffer (PBS pH 7.4 containing 0.5% BSA and 0.02% Azide (eBioscience) were incubated with antibody on ice for 30 mins and washed with DPBS buffer twice. The stained cells were analyzed by BD FACScan (BD, Franklin Lakes, NJ) and the population of cells was recorded.

### RNA isolation

Total RNA from monocyte, piDC, iDC and mDC, and E7 peptide pulsed piDC, iDC and mDC from each donor was isolated, using the Qiagen RNeasy Mini Kit (Qiagen, Inc. Valencia, CA) following the manufacture's instructions. The concentration and quality of the RNA were accessed by NanoDrop spectrophotometer (NanoDrop Technologies, Inc. Wilmington, DE) and Agilent 2100 Bioanalyzer (Agilent Technologies, Inc. Germantown, MD) following the manufacture's instructions. RNA purity is a critical factor for the reliability of microarray data. All total RNA samples had RNA Integrity Number (RIN) higher than 8.6.

### Microarray hybridization

Microarrays were produced in our laboratory, which contained 16, 659 (16K arrays) or 35,035 (35K arrays) transcripts. The arrays contained 70-mer oligonucleotide probes, which were purchased from Operon, Inc. (now Eurofins MWG Operon, Huntsville, AL). The quality of microarray slides was tested as described previously [18].

For study of gene expression profiles of piDC, iDC and mDC as well as their genomic response to E7 peptide stimulation, total RNA isolated from piDC, iDC and mDC and corresponding E7 peptide pulsed piDC, iDC and mDC from four donors (D1–D4) were used as targets, and their mixture of equal amount of monocyte RNA served as a reference (monoRef). 16K arrays were hybridized for this set of experiments. For study of gene expression kinetics in iDCs pulsed with E7 peptide for 2, 10 and 24 hrs, pulsed iDCs were used as targets and non-pulsed served as references. 35K arrays were hybridized for the experiment. Total RNA of 5 µg in 10 µl molecular H_2_O was reverse-transcribed by incubating with 1 µl poly-dT_18_ (1 µg/µl, synthesized by our core facility of CBER/FDA) at 70°C for 2 min, then incubated with 2 µl of 10× first-strand buffer, 2 µl of 20× dNTP (including 10 mM of dATP, dCTP and dGTP, 6 mM of dTTP, and 4 mM of aminoallyl-dUTP), 2 µl of 0.1 M dithiothreitol (DTT), and 1 µl of Affinity Script Multi-Temp Reverse Transcriptase (Stratagene, La Jolla, CA) in a total volume of 20 µl reaction at 42°C for 1 hr as previously described [18], [19]. The cDNA generated was purified with Qiagen MinElute column (Qiagen) according to manufacture's instructions. Reference cDNA and the cDNA of DC samples were swapped labeling with Cy3 and Cy5 (Amersham Biosciences/GE HealthCare, Piscataway, NJ) at room temperature in dark for 1 hr. After incubation, labeled cDNA was purified again with MinElute column. The Cy3-cDNA reference and Cy5-cDNA samples were combined (24 µl) and mixed with 24 µl of SlideHyb Glass Array Hybridization Buffer (Applied Biosystems, Foster City, CA), and hybridized with human arrays in MAUI FL mixer (MAUI Hybridization System Inc., BioMicroSystems, Salt Lake City, UT) overnight (16 hrs). After washing once with 1× SSC (20×, Invitrogen, Grand Island, NY) and 0.05% SDS (10%, Sigma-Aldrich, St. Louis, MO) for 4 min, and twice with 0.1× SSC for 4 min, the arrays were scanned to acquire images.

### Microarray Image Acquisition and Data Analysis

Microarray slides were scanned with a 5-µm resolution at 532 nm (Cy3) and 635 nm (Cy5) using an Axon GenePix 4000B scanner (MDS Analytical Technologies, Sunnyvale, CA). Channels were balanced under laser power of 100% and similar photo-multiplier tube (PMT) setting 640–740 mV. Only uniformly hybridized arrays were accepted. Otherwise, the experiment was repeated. To confirm data reliability, we examined the correlations of hybridization intensity of MonoRef among arrays. All correlations ranged between 0.91 and 0.99.

Images were acquired as .tif files and exported to GenePix Pro 5.0 for data acquisition. Genes/transcripts were annotated through the Gene Array List (.gal file) which was generated with array fabrication and managed by updating with National Center for Biotechnology information (NCBI) genomic database (http://www.ncbi.nlm.nih.gov/) in the Microarray Database (mAdb) at NCI of NIH (https://madb.nci.nih.gov/). Data acquired as .gpr files for data and .jpg files for images were statistically analyzed using mAdb tools and the FDA microarray software – ArrayTrack developed by the National Center for Toxicological Research (http://www.fda.gov/nctr/science/centers/toxicoinformatics/ArrayTrack). After filtering out flagged spots, we normalized the ratio data two ways, a) the Loess fit that minimized local nonlinear biases between the two fluorescence channels, and b) a linear normalization that made the distribution of the log2 intensity ratios to have a median of zero on each slide. Some normalization conditions were set, such as smoothing factor as 0.2, robustness iterations as 3, scaling using geometric mean, and target value as 1000, and the flagged spots were excluded.

Analysis of Variance (ANOVA) was used to search for the significant genes during transition from piDC to iDC and to mDC differentiation stages, the genes/transcripts with an F-value>4 were subjected further pairwise t-tests between stages. Criteria were set as p-value<0.05, excluding bad flagged spots, absolute fold change ≥1.5. Two-class paired Significance Analysis of Microarrays (SAM) was used to compare the gene expression profiles of E7 peptide pulsed piDC, iDC and mDC with corresponding non-pulsed DC samples. Criteria were set as Target False Discovery Rate (FDR)<0.05, excluding bad Flagged spots, absolute fold change ≥1.5. For time course experiments, gene expression was directly compared between E7 pulsed and non-pulsed iDCs from 3 donors (D5–D7). For this purpose, we used mAdb statistical tool available at NCI. We set the criteria as absolute fold change of ≥1.5 fold in all 3 arrays.

The Correlation Matrix function was used to compute between two data sets. Hierarchical Clustering Analysis (HCA) was used to view the correlation of biological repeats based on gene expression. Ingenuity Pathway Analysis (IPA) was used to network the gene biological function.

The Basic Local Alignment Search Tool (BLAST) was used to query features without gene description according to the database of National Center for Biotechnology Information (NCBI, http://blast.ncbi.nlm.nih.gov/Blast.cgi). Blastx was used to query nucleotides in a protein database, and blastp to query translated nucleotides (nt) in proteins database.

### Real-Time Quantitative Polymerase Chain Reaction (qPCR) Assay

Total RNA (1 µg) from iDCs, iDCs pulsed with E7 and control PADRE peptide from two HLA-A2 and two non-HLA-A2 donors was reverse transcribed to cDNA using iScript Reverse Transcription Supermix for RT-qPCR (Bio-Rad Life Science Research, Hercules, CA). The quantification of the expression of NFKBIA was carried out using PrimePCR SYBR Green Assay for human NFKBIA and β-actin using SsoAdvanced SYBR Green Supermix, and CFX96 Touch Deep Well™ Real-Time PCR Detection System (Bio-Rad). The manufacturer's protocols were followed. The level of NFKBIA expression in each sample was normalized to its internal control β-actin. The specificity of each PCR reaction was confirmed by melting curve analyses by CFX manager V3.0 (Bio-Rad) software. The relative quantification of NFKBIA expression in iDCs pulsed with two peptides separately was compared to iDCs.

## Results

### Phenotype of HPV16 E7 class I peptide pulsed human monocytes-derived dendritic cells

**Figure 1:**
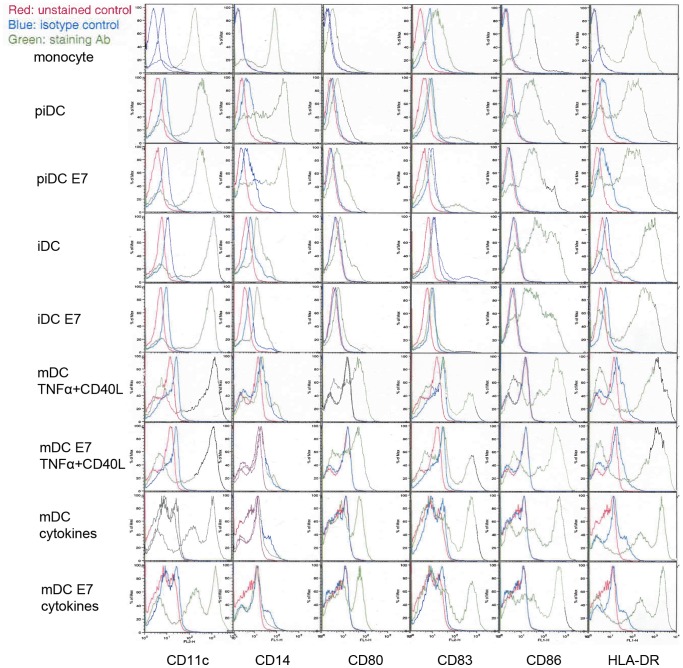
Phenotypes of HPV16 E7 class I peptide pulsed piDC, iDC and mDC. Expression of surface molecules CD11c, CD14, CD83, CD86 and HLA-DR were detected by incubating of monocytes and DCs with FITC-labeled antibodies and analyzed with flow cytometry.

Dendritic cells- piDCs, iDCs and mDCs were generated from human PBMC with different cytokines and under different conditions (**Table 1**). The resultant cells were characterized by fluorescence-activated cell analysis using flow cytometry. As shown in **Fig. 1**, from a representative donor, monocytes cultured for 48 hrs with GM-CSF alone (piDCs) showed a small shift in CD11c expression but expressed similar levels of CD14, CD80, CD83, CD86 and HLA-DR as monocytes. When monocytes were cultured with GM-CSF and IL-4 for 4 days, the resultant cells displayed iDC phenotype by down-regulating monocyte marker CD14 and up-regulating dendritic cell marker CD11c, co-stimulatory molecules CD80 and CD86. When the iDCs were cultured further with CD40 ligand/TNFα or cytokine cocktail for 2 additional days, they began to display mature phenotype by dramatically up-regulating activation marker CD83. The MHC class II molecule (HLA-DR) was also up-regulated in immature and mature DC groups. Interestingly, piDCs, iDCs or mDCs when pulsed with HPV E7-peptide for 2 hrs displayed almost identical phenotype as their non-pulsed states (**Fig. 1**). These results indicate short-term peptide pulsing of DCs does not modulate phenotype that can be appreciated by FACS analysis.

### Gene Expression profiling of DCs

**Figure 2:**
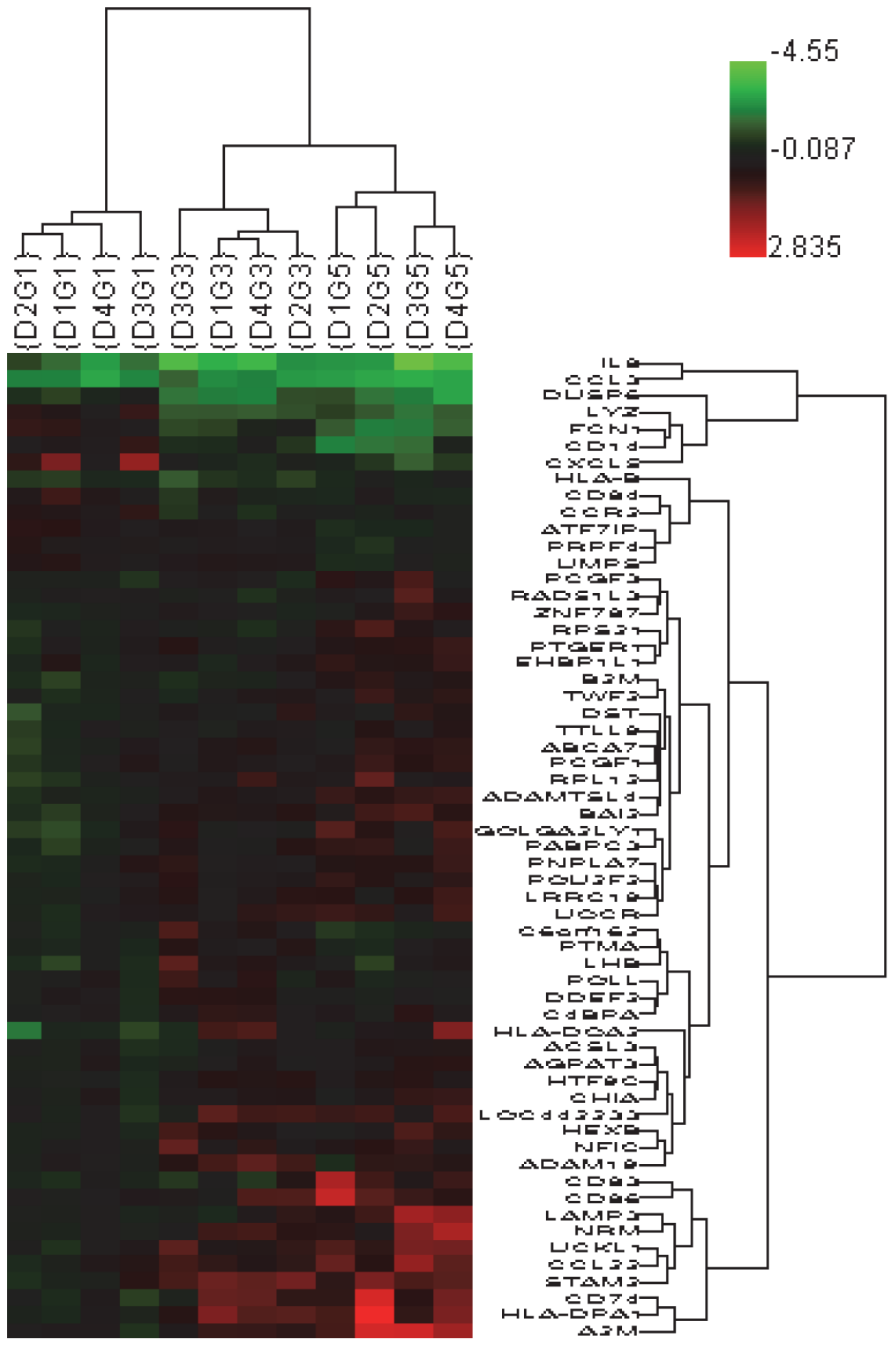
Supervised Hierarchical Clustering Analysis (HCA) presenting a set of significant 59 genes that distinguished three differentiation stages of DCs: piDC (G1), iDC (G3) and mDC (G5). Microarray data of piDC, iDC and mDC from donor D1–D4 were analyzed by ANOVA. Significant genes among the three stages were selected using the criteria set at p-value<0.05 and fold change ≥1.5. Genes with F-ratio>4.7 were presented. Color indicator shows log2 value of the intensity of sample against MonoRef.

We evaluated gene expression profiles of piDCs, iDCs and mDCs from four donors (D1–D4) and found a set of 59 genes that distinguished the three differentiation stages of DCs. The supervised HCA of gene expression showed that piDCs, iDCs and mDCs were grouped together within donors but different between stages of differentiation (**Fig. 2**). Significant gene expression variations between differentiation stages and their description were presented in **Table 2**. Compared to piDCs, iDCs over expressed 26 genes including CD86 and HLA antigens (HLA-DPA1 and –DQA2), and under expressed 7 genes including IL-8 and CD14, which were dominant in monocytes. Compared to iDCs, mDCs over expressed 12 genes including mDC marker genes CD83, CD86 and HLA-B, and under expressed 9 genes including further decrease of CD14. Compared to monocytes, piDCs modulated 32 genes, 17 genes were under expressed representing cytokines IL-6, IL-8, IL-1B, CCL3, CCL14, CCL20, CXCL1. IPA revealed that the 59 genes were involved in immunological and inflammatory diseases, immune cell trafficking, hematological system development and function, and lipid metabolism (**Table 3**).

**Table 2:** Significant Genes during DC differentiation from piDC, iDC to mDC^a^.

mDC/iDC			iDC/piDC			mDC/piDC			piDC/monocyte		
*GeneName*	*Fold* b	*GeneID* c	*GeneName*	*Fold*	*GeneID*	*GeneName*	*Fold*	*GeneID*	*GeneName*	*Fold*	*GeneID*
***Up-reg.***			***Up-reg.***			***Up-reg.***			***Up-reg.***		
A2M	2.70	2	STAM2	2.6	10254	A2M	4.3	2	MRC1	4.5	4360
CD83	2.60	9308	HLA-DQA2	2.6	3118	HLA-DPA1	3.5	3113	LIPA	3.5	3988
LAMP3	2.30	27074	HLA-DPA1	2.5	3113	CD74	3.2	972	NOD1	3.3	10392
RPS21	1.80	6227	LHB	2.2	3972	HLA-DQA2	2.7	3118	FBP1	3.2	2203
B2M	1.70	567	CD74	2.1	972	LAMP3	2.7	27074	TUBA1B	3.2	10376
HLA-B	1.60	3106	ADAM19	1.9	8728	CD83	2.7	9308	FTL	3.1	2512
RAD51L3	1.60	5892	LOC442233	1.9	442233	NRM	2.5	11270	MMP9	3.0	4318
CD86	1.60	942	RPL12	1.9	6136	UCKL1	2.4	54963	GLUL	2.9	2752
CD74	1.50	972	BAI2	1.8	576	GOLGA2LY1	2.4	84559	SPAST	2.5	6683
CCL22	1.50	6367	NRM	1.7	11270	STAM2	2.4	10254	NAGA	2.5	4668
NRM	1.50	11270	DST	1.7	667	CCL22	2.4	6367	CTSB	2.3	1508
GOLGA2LY1	1.50	84559	UCKL1	1.7	54963	CD86	2.3	942	LAMP1	2.1	3916
***Down-reg.***			HEXB	1.7	3074	ADAMTSL4	2.2	54507	IDH1	2.1	3417
CXCL5	0.60	6374	GOLGA2LY1	1.6	84559	BAI2	2.1	576	TGFBI	2.1	7045
ADAM19	0.60	8728	C6orf162	1.6	57150	PABPC3	2.0	5042	TUBB	2.0	2E+05
ATF7IP	0.60	55729	NFIC	1.6	4782	RPL12	2.0	6136	***Down-reg.***		
UMPS	0.60	7372	CCL22	1.6	6367	B2M	2.0	567	DUSP6	0.3	1848
CCL3	0.60	6348	ADAMTSL4	1.6	54507	LOC442233	1.9	442233	ACSL1	0.3	2180
LHB	0.60	3972	HTF9C	1.6	27037	DST	1.9	667	SRGN	0.3	5552
C6orf162	0.50	57150	PABPC3	1.6	5042	RPS21	1.8	6227	CXCL3	0.3	2921
CD14	0.50	929	A2M	1.6	2	ABCA7	1.8	10347	TFPT	0.3	29844
FCN1	0.50	2219	PTMA	1.5	5757	PCGF3	1.7	10336	BTG1	0.3	694
			ABCA7	1.5	10347	UQCR	1.7	10975	CCL7	0.3	6354
			C4BPA	1.5	722	PCGF1	1.7	84759	IL6	0.2	3569
			UQCR	1.5	10975	TWF2	1.7	11344	SOD2	0.2	6648
			CD86	1.5	942	PNPLA7	1.7	375775	CXCL1	0.2	2919
			***Down-reg.***			HEXB	1.7	3074	CCL14	0.2	6358
			CCR2	0.6	729230	PTGER1	1.6	5731	CCL3L3	0.2	4E+05
			CD14	0.6	929	ZNF787	1.6	126208	CCL20	0.2	6364
			FCN1	0.5	2219	EHBP1L1	1.6	254102	CCL3	0.2	6348
			IL8	0.4	3576	POU2F2	1.6	5452	SERPINB2	0.1	5055
			DUSP6	0.4	1848	LRRC19	1.5	64922	IL8	0.1	3576
			CXCL5	0.4	6374	HTF9C	1.5	27037	IL1B	0.1	3553
			LYZ	0.3	4069	TTLL9	1.5	164395			
						CHIA	1.5	27159			
						NFIC	1.5	4782			
						***Down-reg.***					
						IL8	0.4	3576			
						DUSP6	0.4	1848			
						CD14	0.3	929			
						LYZ	0.3	4069			
						CXCL5	0.3	6374			
						FCN1	0.2	2219			

a For these experiments 16K oligonucleotide arrays were used. Microarray data from four donors (D1–D4) were analyzed by ANOVA. Significant genes/transcripts were selected by the criteria of p-value<0.05 and fold ≥1.5. Their F ratio (define here) were >4.7. Significant genes between piDC, iDC and mDC were further selected by pairwise t-test when fold change≥1.5. The piDC/monocyte ratio data were derived from the mean intensity ratio of piDC against monocyte from 4 normalized biological repeat arrays.

b Fold = Fold change, >1.5 means up-regulated, and <0.6 means down-regulated.

c GeneID = GeneEntrezID.

**Table 3:** Functional network analysis of significant genes involved in DC differentiation*.

Gene No.	Top Functions/Molecules in Network
23	*Immunological and Inflammatory Disease*
	B2M, CCL3, CCL22, CD74, CD83, CD84, CD86, CXCL5, HEXB, HLA-B,
	HLA-DPA1, HLA-DQA2, LHB, POU2F2, PTMA, ADAMTSL4,C4BPA,
	FCN1, LAMP3, POLL, PRPF4, RAD51D, ZNF787
17	*Organ Morphology and Development*
	ABCA7, AGPAT3, ASAP2, DST, EHBP1L1, HLA-B, NRM,PABPC3,PCGF1,
	PCGF3, PNPLA7, TRMT2A, TTLL9,TWF2,UCKL1, LHX1,LRRC19
10	*Lipid Metabolism*
	A2M, ACSL3, CD14, DUSP6, IL8, LYZ, NFIC, STAM2, UMPS, UQCR11
8	*Immune Cell Trafficking, Hematological System Development and Function*
	ADAM19, ATF7IP, BAI2, CCR2, CHIA, PTGER1, RPL12, RPS21

* Analyzed by Ingenuity Pathway Analysis (IPA).

We also compared gene expression profiles of mDCs that were maturated by CD40L/TNFα and a cytokine cocktail containing IL1β, IL6, TNFα and PGE_2_. Results showed that CD40L/TNFα (G5) stimulated expression of 52 genes, while the cytokine cocktail (G7) stimulate 108 significant genes in mDC (**Table S1 and Table S2 in File S1**). The increased gene number by cytokine cocktail indicated higher transcription activity of mDCs compared to TNFα and CD40L activated mDCs. Some of these genes overlapped and some were different, indicating different maturation stimuli have different effect on DCs.

### Gene expression profiles of HPV-16 E7 peptide pulsed piDCs, iDCs and mDCs

**Figure 3:**
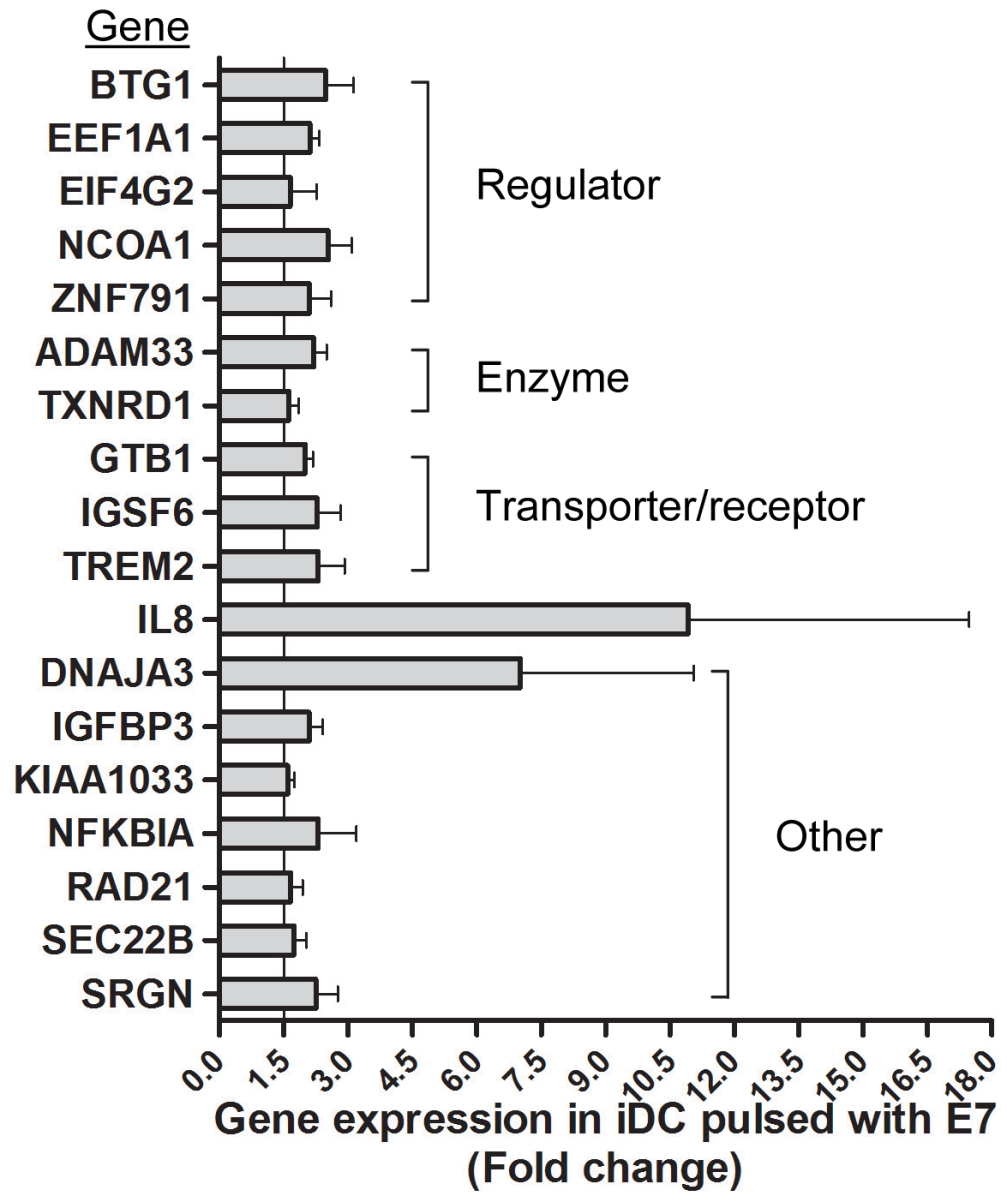
Significant genes of iDCs responding to E7 peptide pulsing. Gene expressions were compared between non-pulsed and pulsed piDCs from donor D1–D4 using two-class paired SAM. Statistical criteria was set as FDR<0.05 and fold change ≥1.5.

We primarily pulsed piDCs, iDCs and mDCs from four donors (D1–D4) with HPV16 E7 (11–20) peptide for 2 hrs and examined gene expression changes using 16K oligonucleotide arrays. By comparing gene expression profiles of the peptide pulsed and non-pulsed DCs, we found one gene in piDC (PPP2R3B), 18 genes in iDC, and no gene in mDC were significantly altered by E7 peptide pulsing (**Fig. 3**). The altered genes were all up-regulated. These data suggest that the differentiated monocytes, mainly iDCs uptake and present antigen, rather than mDCs. Among 18 significantly up-regulated genes, two genes, IL8 and DNAJA3, showed large standard deviations between donors, suggesting that iDC from individual donors respond differently when pulsed with the same antigen. The functional association of the 18 genes was analyzed via IPA software. Four genes (BTG1, EEF1A1, EIF4G2 and NCOA1) belonged to the transcription and translation regulator pathway, 3 genes (ADAM33, IGSF6 and TREM2) associated with trans-membrane activity, 8 genes (ADAM33, GTB1, IGSF6, KIAA1033, RAD21, SEC22B, SRGN and TREM2) were involved in immune response and immunological disease, and four genes (IL8, NFKBIA, SRGN, TREM2) were involved in the antigen presentation.

### Kinetics of gene expression in iDCs pulsed with E7 peptide

To study the kinetics of iDCs responding to peptide pulsing, we pulsed iDCs for 2, 10 and 24 hrs derived from three additional donors' monocytes (D5–D7) and then evaluated for gene expression changes using up-graded microarrays (35K oligonucleotides). At time point 2 hrs, among 52 modulated genes/transcripts, 51 were up-regulated (**Table 4**). We thoroughly searched the gene function in all available resources by using IPA and through feature Report of the microarray database (mAdb) in NCI to RefSeq, RefSeq protein, Gene Bank, GeneCard, EntreZ, and in BioCarta, KEGG and Reactome pathways, as well as Gene Ontology. The up-regulated genes were classified into transcription and translation regulator genes (ANKRD33, BTG1, NCOA1, TEAD4, ZNF791, EEF1A1, EIF4G2 and EIF4E1B); transporter genes (GJB1, SLC25A10 and KPNA7); plasma membrane receptor genes (IGSF6, NCR3, TREM2, NPY2R and GNAO1); plasma membrane bound peptidase and kinase (ADAM33, EPHA1); enzymes (PDE1C, TUBAL3, TXNRD1); growth factor gene PGF; cytokine gene IL8; and others (SNTB1, DNAJA3, KIAA1033, MRPS11, NFKBIA, KIF26B, SEMA6A, SEC22B, CYR61, IGFBP3, SRGN, WFDC5, RAD21 and TNKS1BP1). The remaining 12 were encoding genes with little definition or transcripts with unknown function. The down-regulated gene was OR51B4, a G-protein coupled olfactory receptor.

**Table 4:** Significantly Upregulated Genes in iDCs After E7 Antigen-Peptide Pulsing^a^.

*Gene*	*Fold Change* b		*Function*			*RefSeq*	*EntrezID*	Location & Type
**Upregulated**	2h±SE	Immune	Inflamm-	Signaling	Cell			
		response	tation		survival			
***Regulator***								
ANKRD33	1.92±0.12					NM_182608	341405	Nucleus, transcription
BTG1	1.84±0.27				†	NM_001731	694	Nucleus, transcription
NCOA1	2.40±0.57					NM_003743	8648	Nucleus, transcription
TEAD4	1.68±0.13					NM_003213	7004	Nucleus, transcription
ZNF791	2.05±0.49					NM_153358	163049	Nucleus, transcription
EEF1A1	2.16±0.33						1915	Cytoplasm, translation
EIF4G2	1.87±0.49	†		†		NM_001418	1982	Cytoplasm, translation
EIF4E1B	1.62±0.04					NM_001099408	253314	Cytoplasm, translation
***Transporter***								
GJB1	3.77±0.66			†		NM_001097642	2705	Plasma Membrane
SLC25A10	2.98±0.31					NM_012140	1468	Plasma Membrane
KPNA7	1.56±0.10	†		†		NM_001145715	402569	Cytosol
***Receptor***								
IGSF6	2.29±0.12	†		†		NM_005849	10261	Plasma Membrane
NCR3	2.09±0.07	†	†			NM_001145467	259197	Plasma Membrane
TREM2	2.19±0.30	†	†	†		NM_018965	54209	Plasma Membrane
NPY2R	2.48±0.54			†		NM_000910	4887	Plasma Membrane
GNAO1	2.45±0.43			†		NM_020988	2775	Plasma Membrane, G-protein
***Peptidase, Kinase, Enzyme***								
ADAM33	2.81±0.53						80332	Plasma Membrane, peptidase
EPHA1	1.80±0.04			†		NM_005232	2041	Plasma Membrane, kinase
PDE1C	1.66±0.09			†		NM_001191056	5137	Cytoplasm, phosphodiesterase
TUBAL3	1.48±0.02	†				NM_001171864	79861	Cytoplasm, GTPase
TXNRD1	2.01±0.76				†	NM_003330	7296	Cytoplasm, oxidoreductase
***Growth factor***								
PGF	1.62±0.09			†		NM_002632	5228	Extracellular Space
***Cytokine***								
IL8	1.73±0.17	†	†	†		NM_000584	3576	Extracellular Space
***Other***								
SNTB1	1.63±0.17					NM_021021	6641	Cytoplasm
DNAJA3	2.17±0.19				†	NM_001135110	9093	Cytoplasm
KIAA1033	1.93±0.23					NM_015275	23325	Cytoplasm
MRPS11	2.51±0.06				†	NM_022839	64963	Cytoplasm
NFKBIA	2.37±0.69	†	†	†	†	NM_020529	4792	Cytoplasm
KIF26B	2.17±0.07						55083	Cytoplasm
SEMA6A	1.77±0.05			†	†	NM_020796	57556	Plasma Membrane
SEC22B	1.77±0.22					NM_004892	9554	reticulum membrane
CYR61	1.69±0.21				†	NM_001554	3491	Extracellular Space
IGFBP3	2.07±0.59				†	NM_000598	3486	Extracellular Space
SRGN	1.91±0.13				†	NM_002727	5552	Extracellular Space
WFDC5	2.23±0.24					NM_145652	149708	Extracellular Space
RAD21	2.23±0.12				†	NM_006265	5885	Nucleus
TNKS1BP1	2.45±0.29					NM_033396	85456	Nucleus
C21orf58	1.92±0.33					NM_058180	54058	unknown
C2orf72	2.69±0.29					NM_001144994	257407	unknown
LOC100128374	1.69±0.45					XR_108730	100128374	unknown
LOC100131381	1.63±0.14					XM_001716729	100131381	unknown
LOC729305	1.90±0.15						729305	unknown
PRPE_HUMAN	1.57±0.07							
Unknown	2.88±0.48							
Unknown	1.92±0.22							
Unknown	2.12±0.10							
Unknown	2.08±0.20							
Unknown	1.62±0.09							
Unknown	1.59±0.05							
Unknown	1.63±0.14							
Unknown	2.18±0.28							
**Downregulated**								
OR51B4	0.32±0.02					NM_033179	79339	olfactory receptor

a 35K arrays were used for these experiments. iDCs generated from the monocytes of donor D5–D7 were pulsed with or without E7-peptide for 2, 10 and 24 hrs. Comparison was made between E7 peptide pulsed and non-pulsed iDCs.

b The Fold change represents mean of intensity ratio of E7-pulsed to non-pulsed iDC samples D5–D7, ≥1.5 meaning up-regulation, and ≤0.6 meaning down-regulation. SE is the standard errors. ANOVA showed no significant difference among 3 pulsing time points at p-value<0.5, therefore, significant genes pulsed for 2 hr were presented.

As presented in **Table 4**, 8 genes (EIF4G2, KPNA7, IGSF6, NCR3, TREM2, TUBAL3, IL8, NFKBIA) were involved in immune response, four genes (NCR3, TREM2, IL8, NFKBIA) in inflammation, 13 genes (EIF4G2, GJB1, KPNA7, IGSF6, TREM2, NPY2R, GNAO1, EPHA1, PDE1C, PGF, IL8, SEMA6A and NFKBIA) in signaling, and 10 genes (BTG1, TXNRD1, DNAJA3, MRPS11, NFKBIA, SEMA6A, CYR61, IGFBP3, SRGN and RAD21) in cell survival and cell death. Extending the peptide pulsing time to 10 hrs, the gene expression profiles were similar to 2 hrs pulsed iDCs without any overall statistically significant difference. Continuing pulsing to 24 hrs, there was no further change on overall gene expression except the expression of the NPY2R gene was significantly lower than the other two time points at p-value<0.05. These results indicate that 2 hrs pulsing was sufficient to induce iDC full response at transcription level.

**Figure 4:**
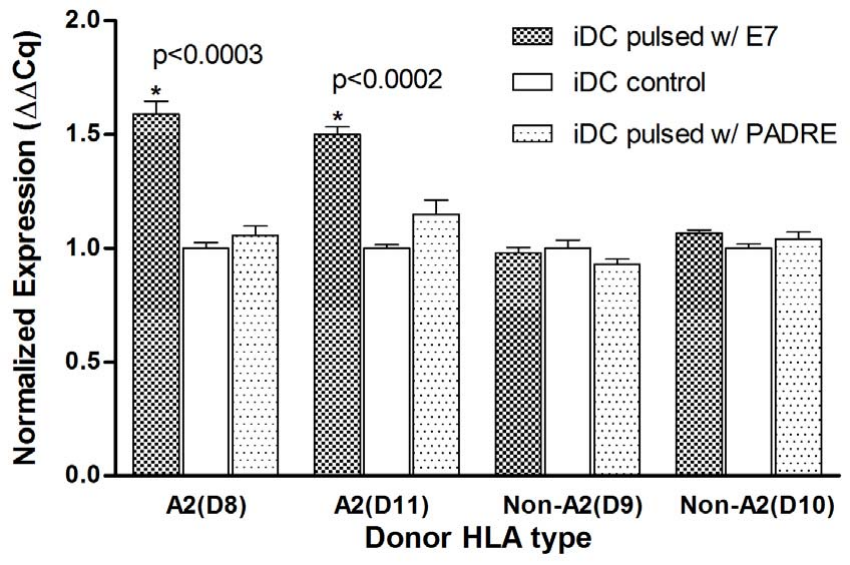
Quantification of NFKBIA gene expression after E7 peptide pulsing of iDCs derived from HLA-A2 disparate donors. iDCs derived from monocytes of two HLA-A2 and two non-HLA-A2 donors (D8–D11) were pulsed with the HLA-A2 binding E7 peptide and a non-natural peptide (PADRE) for 2 hrs. RNA was extracted and reverse transcribed to cDNA. β-actin was used as an internal gene control. Expression of NFKBIA in E7 and PADRE-pulsed iDCs from four different donors were compared to that of native iDCs.

To confirm microarray results and determine the specificity of gene regulation, iDCs generated from HLA-A2 positive and negative donor cells were pulsed with E7 and control PADRE peptides and gene expression of NFKB1A was assessed by Q-PCR. Our results show that E7-peptide pulsing of HLA-A2 positive donor derived iDCs significantly up-regulated NFKBIA gene compared to non-pulsed iDCs (p-value<0.0003 and fold change ≥1.5). In sharp contrast, the E7 peptide pulsing of non-HLA-A2 iDCs showed no regulation of NFKB1A as well as PADRE peptide pulsed iDCs showed no modulation (**Fig. 4**). These results indicate that iDCs response to the E7 stimulation is specific and HLA restricted.

### Bioinformatics of novel genes

We identified six up-regulated features responding to antigen-peptide pulsing but they lacked gene information (**Table S3 in File S1**). Therefore, we queried their potential biological function in the NCBI protein database by using translated BLAST (blastx). The query showed 4 sequences which share similarities with existing proteins and 2 with no significant similarity found. Feature opHsV0400003515 covered 94% with ‘golgi auto-antigen’, opHsV0400005414 covered 68% with ‘cDNA clones with function of inhibiting cancer cell growth’, and H300003055 covered 68% with ‘stAR-related lipid transfer protein’ at <0.001 E-value. We further translated the 2 remaining feature's nucleotide (nt) sequences into amino acid (aa) sequences, and queried their translated residues with NCBI protein database by using protein BLAST (blastp). Within the translated 6 hypothetical peptides of feature opHsV0400007698, 5′3′frame +2 and +3 exhibited partial similarities with ‘trans-membrane and ubiquitin-like domain-containing protein’, ‘multi-drug and toxin extrusion protein’, and ‘ATP-binding cassette sub-family G and C isoform’. Other frames did not bring output or have stop codon in residues. Feature opHsV0400002236 displayed 4 hypothetical proteins, like ‘lysine-specific demethylase’, low-density lipoprotein receptor’, protein bicaudal D’, ‘probable ATP-dependent RNA helicase’, and ‘ADP-ribosyltransferase’ by 4 frames.

## Discussion

By comparing DCs at different stages of differentiation, we identified a set of 59 gene markers that distinguish piDCs, iDCs and mDCs that have been used in cancer vaccine clinical trials. Conventionally, flow cytometry analysis of several surface markers including CD14, CD11c, CD80, CD83, CD86 and HLA-DR are used to characterize DCs. It is known that as PBMCs (as well as piDCs) differentiate to iDC phenotype, iDC cells show down-regulation of CD14 and up-regulation of CD11c, CD86 and HLA-DR. Similarly, as DCs differentiate from iDCs to mDCs, they began to show further up-regulation of CD83, CD86 and HLA-DR [1]. In our study, we also observed these surface markers expressed in DCs. At transcription level, we identified a set of 59 genes that were modulated during DCs differentiation, including CD14 and CD83. These 59 genes may constitute genetic biomarkers of DC differentiation, that can be potentially used to rapidly assess the quality of dendritic cells using nucleic acid based multiplex technology, which is cost effective, efficient and quantitative. Interestingly, we found that despite the fact that piDCs showed no significant changes in the conventional surface markers phenotype when compared to PBMCs, there were significant changes at the gene expression level. These changes may indicate the state of differentiation currently not appreciated by standard cell surface markers.

A large number of studies have reported on the gene expression changes during DC maturation stimulated with different factors. These factors included CD40L [13], [14], [15], TNFα [11], [13], [16], INFγ [13], LPS [8], [10], [15], mixture of INFγ and IL-1β, mixture of LPS and INFγ, mixture of TNFα, IL-1β, LPS and INFγ [8], [20], and cocktails containing IL6, TNFα, IL-1β and PGE_2_ [12], [15]. Simultaneously, various microarray platforms have also been used to profile DCs including cDNA arrays [12], [13], Affymetrix arrays [10], [11], [14], [15], and in-house fabricated oligonucleotide microarrays [8], [20]. Consequently, a large data set has been accumulated in the literature. It has been shown that different stimuli stimulated different gene expression patterns in mDCs. A single factor stimulated a relatively fewer number of genes compared to cytokine cocktails, e.g., CD40L stimulated 40 genes, LPS 619 genes and the combination cocktail 1116 genes after 48 hr culture of mDCs [15]. Recently, some studies have identified as much as 9576 genes that were modulated in mDCs [8], [20]. Moschella *et al.*, used a single cytokine TNFα, CD40L or INFγ as stimuli and found that they induced different gene expression patterns in mDCs, suggesting important functional differences among the DC populations [13]. Han *et al.*, used three combinations, LPS plus INFγ, INFγ plus IL-1β, and a cocktail that contained LPS, INFγ, IL-1β and TNFα as DC maturation stimuli, and found that these 3 combinations had no difference in terms of expression of co-stimulatory molecules CD80, CD83 and CD86, HLA-DR, chemokine receptor CCR7, and interleukin IL-12p70and IL-10 [8]. Jin *et al.*, recommended CCL5, CCR7 and CD83 as particularly good potency biomarker candidates that were selected by evaluating 2370 significant modulated genes for maturation [20]. Although the modulated gene number varied, in most publications, the activated pathways remained the same. These common pathways are cell signaling, cell adhesion and lipid metabolism [8], [11], [20].

In our study, we also compared gene expression profiles of mDCs stimulated by a cytokine cocktail (IL-1β, IL6, TNFα and PGE_2_) with mDCs that stimulated by CD40L and TNFα. The cytokine cocktail induced 108 genes in mDCs *vs.* 52 genes induced with CD40L and TNFα. Consistent with previous studies [11], [12], [14], [15], the results show that the cytokine cocktail supplies stronger stimulation that induced more modulated gene during the process of DC maturation than CD40L/TNFα. The modulated genes were categorized into cell signaling, immune response and lipid metabolism that are very similar with previous publications [11]. As CD83, CD86, LAMP3 and DUPS6 up-modulated, and CD14 and CCR2 down-modulated, our results agree with other reports in which CD40L or TNFα were used as stimuli [10], [11], [13]. Further studies are needed to determine the significance of these stimuli in DC function.

The HPV-16 E7 HLA-A2 (11–20) peptide has been used in clinical trials for the treatment of patients with cervical neoplasms. The vaccination with this peptide, albeit administered directly or pulsed on DCs in these clinical trials has shown series of immune and clinical benefits [21], [22], [23]. However, the molecular profile of peptide pulsed DCs were not known. Hence, we tested the effect of the peptide pulsing on the gene expression profile of DCs; whether differentiation stages of DCs affect the effect; and whether peptide-antigen pulsed DC can rapidly be assessed by gene expression profiling. In our experiments, when piDC, iDC and mDC were pulsed with E7 peptide for 2 hrs, no phenotypic changes were observed by flow cytometry analysis. However, at transcription level, iDCs, but not mDCs, responded to the peptide-pulsing by up-regulating a set of genes. This set of genes we identified is apparently related to iDC antigen uptake, processing and presentation (ADAM33, KIF26B, MRPS11, NPY2R, SLC25A10, TNKS1BP1, TREM2, and ZNF791). Thus, our study may be the first evaluation of gene expression of E7-peptide pulsing of DC for immunotherapy.

Gene NFKBIA attracted a special attention since it was highly expressed at all three time points after antigen pulsing (at 2, 10 and 24 hrs). NFKBIA functions as a ‘nuclear factor of kappa light polypeptide gene enhancer’ promoting IL-6 and TNF-α synthesis. It has been shown that human tumor antigen MUC1, when pulsed into iDC, produced inflammatory cytokines IL-6 and TNF-α [24], [25], which in turn induced a strong MUC1-specific CD8+ T-cell response [26]. Therefore, it is likely that the E7 peptide pulsed DCs will generate a strong CD8+ T cell response. Interestingly, E7 up-regulated four pro-apoptosis genes (BTG1, SEMA6A, IGFBP3 and SRGN) in iDCs indicating that antigen pulse selectively depletes some population of iDCs. However, NFKBIA, a strong negative regulator of apoptosis, and 3 other genes (MRPS11, RAD21 and TXNRD1) involved in DNA damage repair were up-regulated that supported cell survival. In addition, another gene DNAJA3 also involved in cell survival was up-regulated at all three time points. These counteracting mechanisms may allow iDC to survive and present antigen. Furthermore, 4 genes (EPHA1, PGF, IL8 and CYR61) involved in cell adhesion were also up regulated at all three time points indicating significance of these genes in iDC antigen presentation, interaction with T cells and biology.

The mere fact that only iDC showed changes in the gene expression profile after E7 pulsing indicates that these changes are probably secondary to the antigen uptake process, since iDC is the only DC type amongst the three that is capable of antigen uptake in addition to presentation. We confirmed the response of iDCs by Q-PCR, which also demonstrated that E7 induced NFKB1A gene expression is HLA restricted since iDCs derived from non-HLA-A2 donors did not respond to E7 stimulation. Furthermore, the upregulation of NFKBIA expression in iDCs was specific as PADRE-peptide pulse did not induce gene expression. Further studies are needed to determine whether the gene expression markers identified by the HPV16 E7 antigen-peptide are unique to that sequence or similar when pulsed with different antigens.

It is important to note that we identified 6 potential novel genes that were induced by antigen pulsing of iDC, as gene information and description of these putative genes is unknown in the current NCBI nucleotide database. When their potential functions were queried by BCBI Blastx, we found that three of these genes have high similarities with ‘golgi auto-antigen’, ‘cDNA clones with function of inhibiting cancer cell growth’, and ‘stAR-related lipid transfer protein’ with very low e-values and high max scores. The other 2 feature's lacking similarities with the nucleotide (nt) database were further translated into amino acid (aa) sequences, and their translated residues were queried with the NCBI protein database by blastp. These analyses showed similarities with ‘transmembrane and ubiquitin-like domain-containing protein’, ATP-binding cassette sub-family G and C isoforms’, ‘lysine-specific demethylase’, and low-density lipoprotein receptor’. Although the exact identity and significance of these novel genes are still not known, they may have an important role in antigen presentation and immune response.

Taken together, our results indicate that peptide-pulsing induces changes in iDC which may be important for optimal functioning of DCs *in vivo*. In addition, we have identified novel gene markers of antigen uptake and presentation that can be rapidly assessed and when correlated with function may serve as a surrogate marker of potency. Further studies will examine other peptide antigens and analyze similarity in gene expression profile.

## Supporting Information

File S1 **Contents include: Table S1. Significant genes in mDC induced by CD40L/TNFα compared with piDC. Table S2. Significant genes in mDC induced by cytokine cocktail compared with piDC. Table S3. Bioinformation of novel genes.**
(XLS)

## References

[1] Banchereau J, Steinman RM (1998) Dendritic cells and the control of immunity. Nature 392: 245–252. 9521319 10.1038/32588

[2] Soumelis V, Liu YJ (2006) From plasmacytoid to dendritic cell: morphological and functional switches during plasmacytoid pre-dendritic cell differentiation. Eur J Immunol 36: 2286–2292. 16892183 10.1002/eji.200636026

[3] Banchereau J, Palucka AK (2005) Dendritic cells as therapeutic vaccines against cancer. Nat Rev Immunol 5: 296–306. 15803149 10.1038/nri1592

[4] Schlom J (2012) Therapeutic cancer vaccines: current status and moving forward. J Natl Cancer Inst 104: 599–613. 22395641 10.1093/jnci/djs033 PMC3328421

[5] Sallusto F, Lanzavecchia A (1994) Efficient presentation of soluble antigen by cultured human dendritic cells is maintained by granulocyte/macrophage colony-stimulating factor plus interleukin 4 and downregulated by tumor necrosis factor alpha. J Exp Med 179: 1109–1118. 8145033 10.1084/jem.179.4.1109 PMC2191432

[6] Caux C, Dezutter-Dambuyant C, Schmitt D, Banchereau J (1992) GM-CSF and TNF-alpha cooperate in the generation of dendritic Langerhans cells. Nature 360: 258–261. 1279441 10.1038/360258a0

[7] Jonuleit H, Kuhn U, Muller G, Steinbrink K, Paragnik L, et al (1997) Pro-inflammatory cytokines and prostaglandins induce maturation of potent immunostimulatory dendritic cells under fetal calf serum-free conditions. Eur J Immunol 27: 3135–3142. 9464798 10.1002/eji.1830271209

[8] Han TH, Jin P, Ren J, Slezak S, Marincola FM, et al (2009) Evaluation of 3 clinical dendritic cell maturation protocols containing lipopolysaccharide and interferon-gamma. J Immunother 32: 399–407. 19342965 10.1097/CJI.0b013e31819e1773 PMC2832587

[9] Tsukui T, Hildesheim A, Schiffman MH, Lucci J 3rd, Contois D, et al (1996) Interleukin 2 production in vitro by peripheral lymphocytes in response to human papillomavirus-derived peptides: correlation with cervical pathology. Cancer Res 56: 3967–3974. 8752165

[10] Lindstedt M, Johansson-Lindbom B, Borrebaeck CA (2002) Global reprogramming of dendritic cells in response to a concerted action of inflammatory mediators. Int Immunol 14: 1203–1213. 12356685 10.1093/intimm/dxf082

[11] Le Naour F, Hohenkirk L, Grolleau A, Misek DE, Lescure P, et al (2001) Profiling changes in gene expression during differentiation and maturation of monocyte-derived dendritic cells using both oligonucleotide microarrays and proteomics. J Biol Chem 276: 17920–17931. 11279020 10.1074/jbc.M100156200

[12] Dietz AB, Bulur PA, Knutson GJ, Matasic R, Vuk-Pavlovic S (2000) Maturation of human monocyte-derived dendritic cells studied by microarray hybridization. Biochem Biophys Res Commun 275: 731–738. 10973791 10.1006/bbrc.2000.3372

[13] Moschella F, Maffei A, Catanzaro RP, Papadopoulos KP, Skerrett D, et al (2001) Transcript profiling of human dendritic cells maturation-induced under defined culture conditions: comparison of the effects of tumour necrosis factor alpha, soluble CD40 ligand trimer and interferon gamma. Br J Haematol 114: 444–457. 11529869 10.1046/j.1365-2141.2001.02953.x

[14] Tureci O, Bian H, Nestle FO, Raddrizzani L, Rosinski JA, et al (2003) Cascades of transcriptional induction during dendritic cell maturation revealed by genome-wide expression analysis. FASEB J 17: 836–847. 12724343 10.1096/fj.02-0724com

[15] Messmer D, Messmer B, Chiorazzi N (2003) The global transcriptional maturation program and stimuli-specific gene expression profiles of human myeloid dendritic cells. Int Immunol 15: 491–503. 12663679 10.1093/intimm/dxg052

[16] McIlroy D, Tanguy-Royer S, Le Meur N, Guisle I, Royer PJ, et al (2005) Profiling dendritic cell maturation with dedicated microarrays. J Leukoc Biol 78: 794–803. 15961579 10.1189/jlb.0105029

[17] Cools N, Ponsaerts P, Lenjou M, Nijs G, Van Bockstaele DR, et al (2006) Sensitive detection of human papillomavirus type 16 E7-specific T cells by ELISPOT after multiple in vitro stimulations of CD8+ T cells with peptide-pulsed autologous dendritic cells. Mol Cancer 5: 49. 17067378 10.1186/1476-4598-5-49 PMC1634756

[18] Yang AX, Mejido J, Bhattacharya B, Petersen D, Han J, et al (2006) Analysis of the quality of contact-pin fabricated oligonucleotide microarrays. Mol Biotechnol 34: 303–315. 17284778 10.1385/MB:34:3:303

[19] Han J, Lee H, Nguyen NY, Beaucage SL, Puri RK (2005) Novel multiple 5′-amino-modified primer for DNA microarrays. Genomics 86: 252–258. 15949917 10.1016/j.ygeno.2005.04.009

[20] Jin P, Han TH, Ren J, Saunders S, Wang E, et al (2010) Molecular signatures of maturing dendritic cells: implications for testing the quality of dendritic cell therapies. J Transl Med 8: 4. 20078880 10.1186/1479-5876-8-4 PMC2841589

[21] Murakami M, Gurski KJ, Marincola FM, Ackland J, Steller MA (1999) Induction of specific CD8+ T-lymphocyte responses using a human papillomavirus-16 E6/E7 fusion protein and autologous dendritic cells. Cancer Res 59: 1184–1187. 10096544

[22] Santin AD, Hermonat PL, Ravaggi A, Chiriva-Internati M, Zhan D, et al (1999) Induction of human papillomavirus-specific CD4(+) and CD8(+) lymphocytes by E7-pulsed autologous dendritic cells in patients with human papillomavirus type 16- and 18-positive cervical cancer. J Virol 73: 5402–5410. 10364287 10.1128/jvi.73.7.5402-5410.1999 PMC112596

[23] Welters MJ, Kenter GG, Piersma SJ, Vloon AP, Lowik MJ, et al (2008) Induction of tumor-specific CD4+ and CD8+ T-cell immunity in cervical cancer patients by a human papillomavirus type 16 E6 and E7 long peptides vaccine. Clin Cancer Res 14: 178–187. 18172269 10.1158/1078-0432.CCR-07-1880

[24] Carlos CA, Dong HF, Howard OM, Oppenheim JJ, Hanisch FG, et al (2005) Human tumor antigen MUC1 is chemotactic for immature dendritic cells and elicits maturation but does not promote Th1 type immunity. J Immunol 175: 1628–1635. 16034102 10.4049/jimmunol.175.3.1628

[25] Cascio S, Zhang L, Finn OJ (2011) MUC1 protein expression in tumor cells regulates transcription of proinflammatory cytokines by forming a complex with nuclear factor-kappaB p65 and binding to cytokine promoters: importance of extracellular domain. J Biol Chem 286: 42248–42256. 22021035 10.1074/jbc.M111.297630 PMC3234962

[26] Beatty P, Ranganathan S, Finn OJ (2012) Prevention of colitis-associated colon cancer using a vaccine to target abnormal expression of the MUC1 tumor antigen. Oncoimmunology 1: 263–270. 22737601 10.4161/onci.18950 PMC3382848

